# Medical Colleges

**Published:** 1875-08

**Authors:** 


*Medical Colleges.*

The time is at hand for medical students to seek a college for the attainment of the most valuable scientific education. Being the organ of no particular college, THE RECORD feels free in advising every friend or preceptor of students to advise them to be extremely careful in their selection of a college. Let them attend only such colleges as are eminent for the thoroughness and scientific attainments of its faculty, and the completeness of its means and apparatus.

The influence of their college education is permanent upon every student; therefore such influence should be of the most perfect kind, in order to insure to him the success which he is striving for in his profession. "As the twig is bent the tree is inclined." Let students carefully avoid so-called colleges which are such only in name, and whose existence is merely for the purpose of advertising a ring of men of more ambition than merit.

There are such in every country, and their baneful influence upon our profession are painfully evident. This much, as journalists, we feel it to be our duty to say, but must ask our estimable friend, Dr. D. A. W., to excuse us for declining to publish his letter of recent date, exposing the means used by the Dean of a certain institution to advertise his college, and induce students to attend it. Men who wish to monopolize everything seldom succeed long. The notoriety they give themselves rarely fails to destroy the little reputation and character they may deserve. The convention representing the colleges must attend to such departures. All that you and your medical friends can do is to see to it that your students are not controlled by such influences. P.

